# Supplementary material for: Long-Term Outcomes of a Randomized Study of Neoadjuvant Induction Dual HER2 Blockade with Trastuzumab and Lapatinib Followed by Weekly Paclitaxel Plus Dual HER2 Blockade for HER2-Positive Primary Breast Cancer (Neo-Lath Study)
Source: Cancers (Basel). 2021 Aug 9;13(16):4008. doi: 10.3390/cancers13164008 (PMC8394774; doi:10.3390/cancers13164008)
Supplement: Supplementary file 1 [file cancers-13-04008-s001.zip › cancers-1289943-supplementary.pdf]

cancers

MDPI

Long-Term Outcomes of a Randomized Study of Neoadjuvant Induction Dual HER2 Blockade with Trastuzumab and Lapatinib Followed by Weekly Paclitaxel Plus Dual HER2 Blockade for HER2-Positive Primary Breast Cancer (Neo-Lath Study)

Eriko Tokunaga, Norikazu Masuda, Naohito Yamamoto, Hiroji Iwata, Hiroko Bando, Tomoyuki Aruga, Shoichiro Ohtani, Tomomi Fujisawa, Toshimi Takano, Kenichi Inoue, Nobuyasu Suganuma, Masahiro Takada, Kenjiro Aogi, Kenichi Sakurai, Hideo Shigematsu, Katsumasa Kuroi, Hironori Haga, Shinji Ohno, Satoshi Morita and Masakazu Toi

Supplementary Material

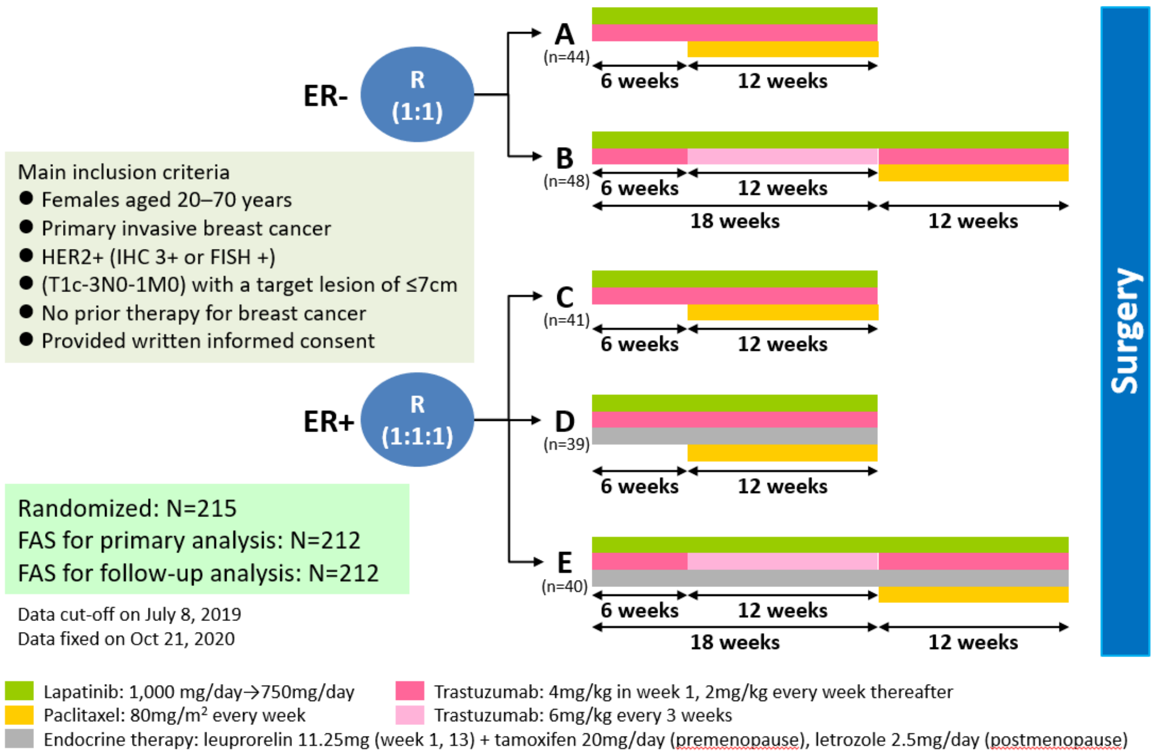

Figure S1. Study design of the Neo-LaTH study. ER, estrogen receptor; FAS, full analysis set; FISH, fluorescence in situ hybridization; HER, human epidermal growth factor receptor; IHC, immunohistochemistry.

A

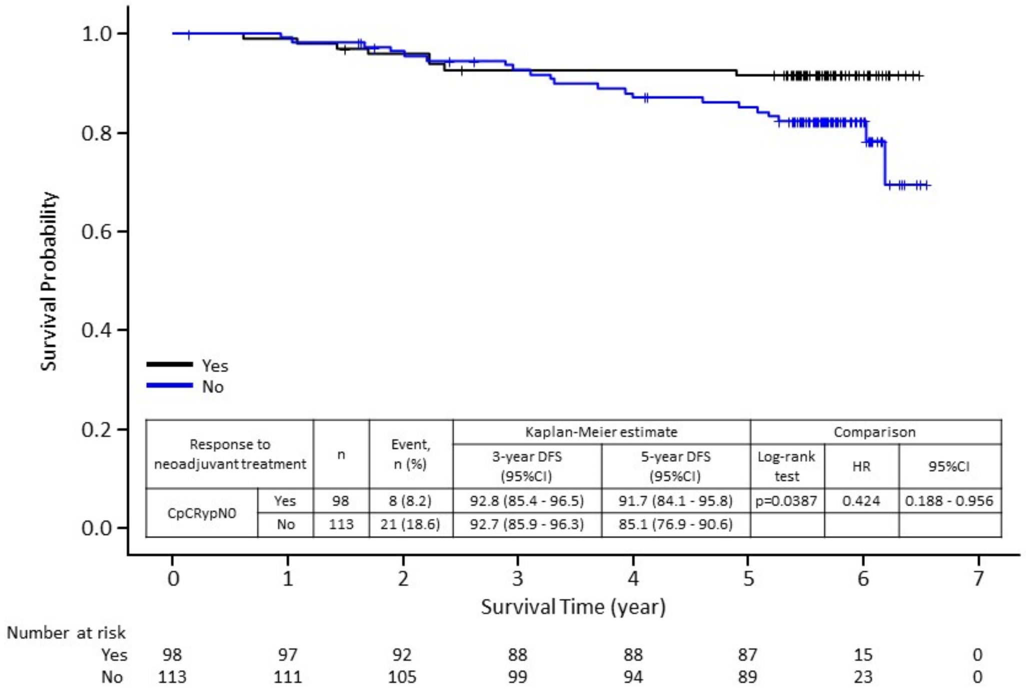
1.0
0.8
0.6
0.4
0.2
0.0
Survival Probability
Yes
No
Response to neoadjuvant treatment
n
Event, n (%)
Kaplan-Meier estimate
3-year DFS (95%CI)
5-year DFS (95%CI)
Comparison
Log-rank test
HR
95%CI
CpCRypN0
Yes 98 8 (8.2) 92.8 (85.4 - 96.5) 91.7 (84.1 - 95.8) p=0.0387 0.424 0.188 - 0.956
No 113 21 (18.6) 92.7 (85.9 - 96.3) 85.1 (76.9 - 90.6)
0 1 2 3 4 5 6 7
Survival Time (year)
Number at risk
Yes 98 97 92 88 88 87 15 0
No 113 111 105 99 94 89 23 0

B

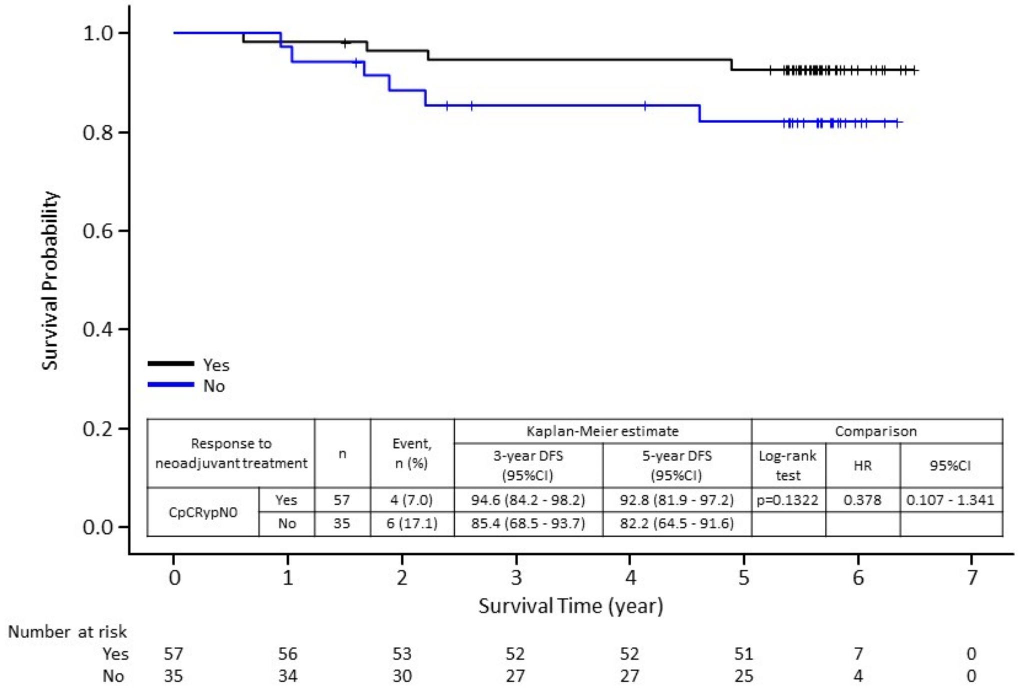
1.0
0.8
0.6
0.4
0.2
0.0
Survival Probability
Yes
No
Response to neoadjuvant treatment
n
Event, n (%)
Kaplan-Meier estimate
3-year DFS (95%CI)
5-year DFS (95%CI)
Comparison
Log-rank test
HR
95%CI
CpCRypN0
Yes 57 4 (7.0) 94.6 (84.2 - 98.2) 92.8 (81.9 - 97.2) p=0.1322 0.378 0.107 - 1.341
No 35 6 (17.1) 85.4 (68.5 - 93.7) 82.2 (64.5 - 91.6)
0 1 2 3 4 5 6 7
Survival Time (year)
Number at risk
Yes 57 56 53 52 52 51 7 0
No 35 34 30 27 27 25 4 0

C

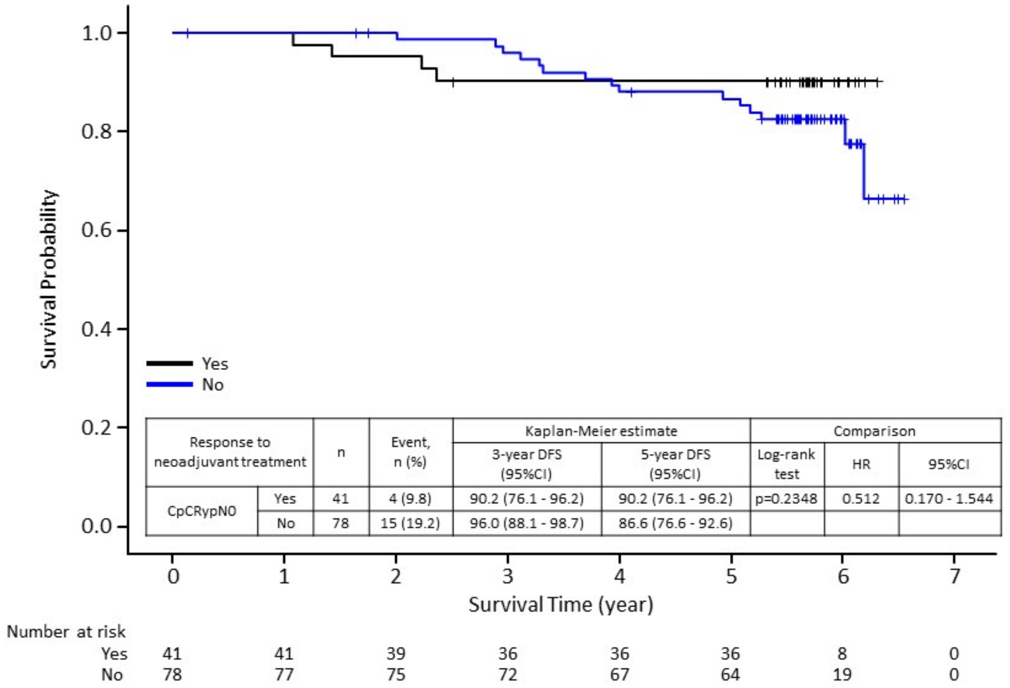

Response to neoadjuvant treatment		n	Event, n (%)	Kaplan-Meier estimate		Comparison		
				3-year DFS (95%CI)	5-year DFS (95%CI)	Log-rank test	HR	95%CI
CpCRypN0	Yes	41	4 (9.8)	90.2 (76.1 - 96.2)	90.2 (76.1 - 96.2)	p=0.2348	0.512	0.170 - 1.544
	No	78	15 (19.2)	96.0 (88.1 - 98.7)	86.6 (76.6 - 92.6)			

Figure S2. Kaplan–Meier curves of disease-free survival (DFS) stratified by response to neoadjuvant treatment (with or without CpCRypN0): A. all patients (*n* = 211), B. estrogen receptor (ER)-negative patients (*n* = 92), and C. ER-positive patients (*n* = 119). CI, confidence interval; CpCRypN0, comprehensive pathological complete response with a pathologically negative axilla; HR, hazard ratio.

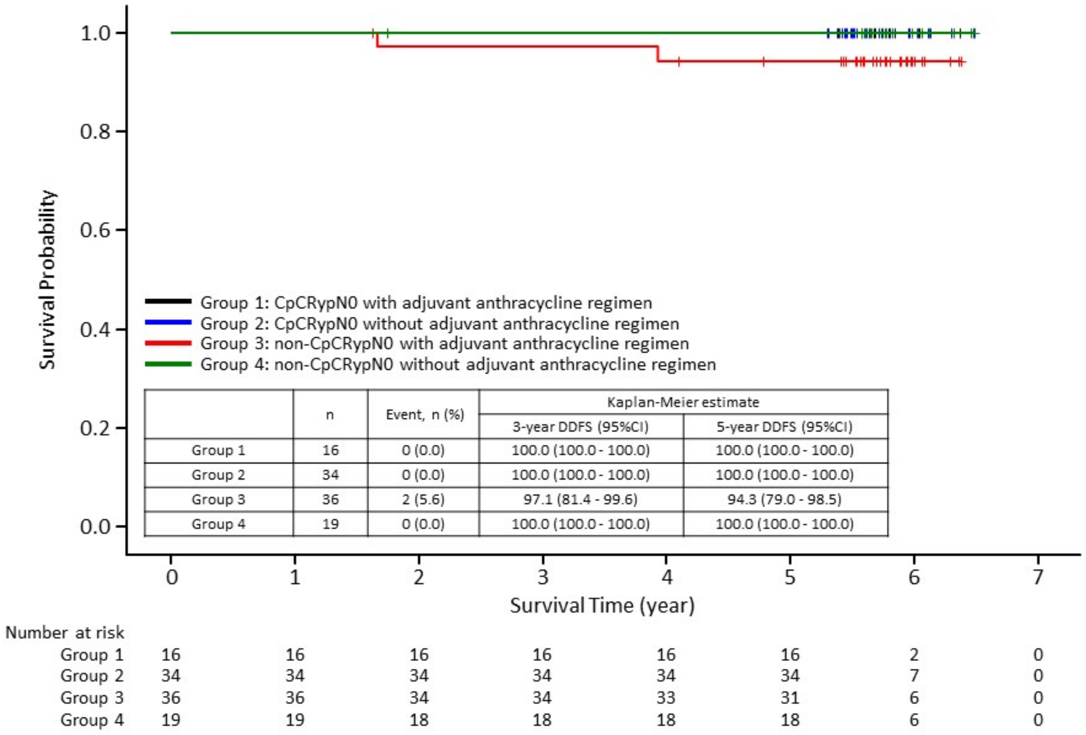

	n	Event, n (%)	Kaplan-Meier estimate	
			3-year DDFS (95%CI)	5-year DDFS (95%CI)
Group 1	16	0 (0.0)	100.0 (100.0 - 100.0)	100.0 (100.0 - 100.0)
Group 2	34	0 (0.0)	100.0 (100.0 - 100.0)	100.0 (100.0 - 100.0)
Group 3	36	2 (5.6)	97.1 (81.4 - 99.6)	94.3 (79.0 - 98.5)
Group 4	19	0 (0.0)	100.0 (100.0 - 100.0)	100.0 (100.0 - 100.0)

Figure S3. Kaplan–Meier curves of distant disease-free survival (DDFS) in patients with T1cT2N0 stratified by response to neoadjuvant treatment (with or without CpCRypN0) and with or without use of adjuvant anthracycline in all patients (*n* = 105). CI, confidence interval. CpCRypN0, comprehensive pathological complete response with a pathologically negative axilla.
